# From Oral Health to Obstetric Outcomes: A Comprehensive Review of Periodontal Disease and Its Implications for Preeclampsia

**DOI:** 10.7759/cureus.62995

**Published:** 2024-06-23

**Authors:** Medhavi Sharma, Urmila Sunda, Pankhuri Dubey, Hard Tilva

**Affiliations:** 1 Obstetrics and Gynecology, Assistant Professor, All India Institute of Medical Sciences, Rajkot, IND; 2 Obstetrics and Gynecology, Jawaharlal Nehru Medical College, Datta Meghe Institute of Higher Education and Research, Wardha, IND; 3 Obstetrics and Gynecology, All India Institute of Medical Sciences, Rajkot, IND

**Keywords:** inflammation, oral health, obstetric outcomes, pregnancy, pre-eclampsia, periodontal disease

## Abstract

Periodontal disease and preeclampsia (PE) are both significant health concerns with implications for maternal and fetal well-being. Emerging evidence suggests a potential association between these two conditions, prompting increased interest in understanding their relationship and clinical implications. This comprehensive review examines the current literature on periodontal disease and PE, focusing on epidemiological evidence, proposed mechanistic pathways, and clinical implications. Epidemiological studies consistently demonstrate an increased risk of PE among pregnant individuals with periodontal disease, independent of traditional risk factors. Proposed mechanisms linking periodontal disease to PE include systemic inflammation, endothelial dysfunction, and immune dysregulation. The implications for research include the need for well-designed prospective studies and randomized controlled trials to elucidate further the mechanisms underlying the association and evaluate the effectiveness of periodontal interventions in preventing PE. Clinicians should be aware of the potential link between periodontal disease and PE and consider screening pregnant individuals for periodontal disease as part of routine prenatal care. Interdisciplinary collaboration between obstetricians and periodontists may be beneficial in managing pregnant individuals with periodontal disease to mitigate the risk of PE. By addressing these research gaps, we can further understand the relationship between oral health and obstetric outcomes and develop evidence-based strategies to improve maternal and fetal health.

## Introduction and background

Periodontal disease is a chronic inflammatory condition affecting the supporting structures of the teeth, including the gums, periodontal ligament, and alveolar bone. It is characterized by gingival inflammation, periodontal pocket formation, and loss of connective tissue attachment to the tooth. The primary etiological factor is the accumulation of bacterial plaque on the tooth surfaces, leading to an inflammatory response by the host immune system [1]. Preeclampsia (PE) is a multisystem disorder characterized by hypertension and proteinuria that typically arises after 20 weeks of gestation. It is one of the leading causes of maternal and perinatal morbidity and mortality worldwide. PE is associated with a range of maternal complications, including eclampsia, stroke, renal failure, and hepatic dysfunction, as well as adverse fetal outcomes such as intrauterine growth restriction and preterm birth [2].

Emerging evidence suggests a potential link between periodontal disease and PE. Several studies have reported an association between periodontal inflammation and an increased risk of developing PE, independent of traditional risk factors. The mechanisms underlying this association are thought to involve systemic inflammation, endothelial dysfunction, and immune dysregulation, although the precise pathways remain fully elucidated [3]. This review aims to provide a comprehensive overview of the relationship between periodontal disease and PE. It will examine the epidemiological evidence supporting this association, discuss potential mechanisms underlying the link, and explore the clinical implications for obstetric practice. Furthermore, the review will highlight current gaps in knowledge and identify areas for future research to understand better and manage the interplay between oral health and obstetric outcomes.

## Review

Periodontal disease: etiology and pathogenesis

Definition and Types of Periodontal Disease

**Figure 1:**
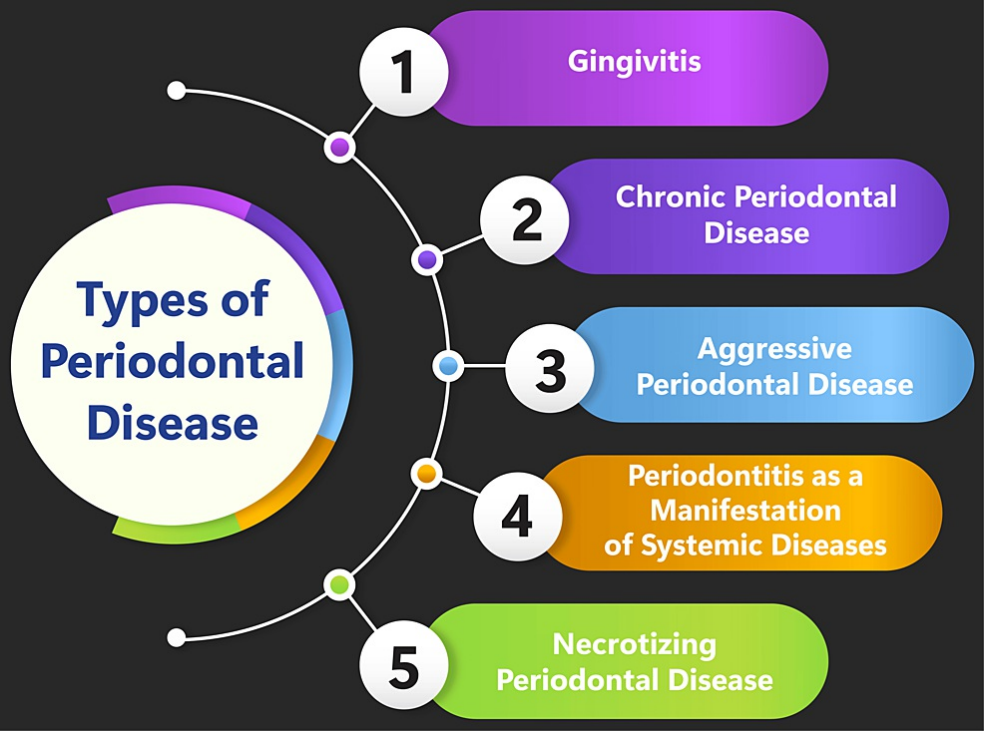
Types of periodontal disease. Image credit: Medhavi Sharma.

Periodontal disease, or periodontitis or gum disease, is a progressive condition that impacts the supportive and surrounding tissues of the gums and the underlying jawbone. Its primary cause lies in the presence of bacteria in plaque, triggering inflammation and gum infection. Left untreated, periodontal disease can lead to tooth instability and even loss, rendering it the foremost cause of tooth loss among adults in the developed world [1]. Gingivitis represents the mildest and most prevalent form of periodontal disease, identifiable by red, swollen gums that easily bleed. This condition is reversible with professional treatment and adherence to good oral hygiene practices such as regular brushing and flossing [4]. Chronic Periodontal Disease, the most prevalent form, typically afflicts individuals aged 45 and older. It involves inflammation beneath the gum line and gradual deterioration of the supporting tissues surrounding the teeth. While it cannot be entirely cured, treatment strategies can effectively halt its progression [5]. Aggressive Periodontal Disease is characterized by rapid gum attachment loss and bone destruction. It advances swiftly and can cause substantial damage if not promptly addressed [6]. Periodontitis as a Manifestation of Systemic Diseases is linked to systemic conditions like diabetes, heart disease, and respiratory ailments. This form involves inflammation within the supportive tissues of the teeth, progressive attachment and bone loss, and the formation of pockets [7]. The necrosis of gingival tissues, the periodontal ligament, and alveolar bone typifies Necrotizing Periodontal Disease. It is frequently observed in individuals with systemic conditions like HIV infection, malnutrition, and immunosuppression [8]. Understanding the various forms of periodontal disease and their associated risk factors is pivotal for effective diagnosis and treatment. Moreover, recognizing the systemic implications of periodontal disease underscores the significance of comprehensive oral health care in managing overall health and well-being. Types of periodontal disease are shown in Figure 1.

Microbiology of Periodontal Disease

The microbiology of periodontal disease entails an intricate interplay between Gram-negative bacteria and the host's inflammatory response, culminating in detrimental alterations in the periodontal tissues. Prominent bacteria associated with periodontal disease include Porphyromonas gingivalis, Bacteroides forsythus, and Actinobacillus actinomycetemcomitans, which possess virulence factors contributing to tissue degradation [9]. These bacteria are implicated in the onset and advancement of periodontal diseases, manifesting symptoms such as bleeding gums, halitosis, gum inflammation, and tooth mobility [10]. The pathogenesis of periodontal disease involves the buildup of microbial plaque, acute inflammatory reactions, and subsequent tissue breakdown, resulting in conditions like gingivitis and periodontitis [10,11]. Studies underscore the polymicrobial nature of periodontal diseases, with diverse bacterial species interacting within biofilms to induce inflammation and tissue impairment, underscoring the necessity of comprehending the microbiological dimensions for efficacious management and treatment of periodontal diseases [9].

Immunological Factors

Immunological factors are pivotal in the onset and advancement of periodontal disease. The host's immune response to periodontopathic bacteria is a critical determinant in the degradation of soft and hard tissues seen in chronic and aggressive periodontitis [12]. The immune response elicited by periodontal disease extends beyond the presence of bacteria in plaque, encompassing a spectrum of immune mechanisms contributing to the disease's progression [12]. It is widely acknowledged that the host immune system's reaction to periodontopathic pathogens constitutes a significant component in the pathogenesis of periodontitis, marked by the intricate interplay between these bacteria and the host's immune system [12]. This interplay can provoke an imbalance in the immune response, tipping the scales in favor of disease progression [13]. Comprehending the immunological intricacies of periodontal disease is imperative for devising effective therapeutic strategies. This includes interventions aimed at immunomodulation and mitigating chronic oxidative stress, which are vital avenues for developing treatments that can effectively halt or reverse the progression of periodontal disease [13].

Genetic Predisposition

Genetic predisposition holds considerable sway in the onset and progression of periodontal disease. Those with a genetic inclination toward periodontitis exhibit gene expressions that shape their immune system's response to harmful bacteria in the gums and arteries, impacting the severity and pace at which the disease manifests [14]. Extensive research underscores genetics as a major risk factor influencing the likelihood of developing periodontal disease, alongside factors such as bacterial biofilms, lifestyle choices, systemic conditions, and other miscellaneous factors [14]. Numerous studies have pinpointed various genetic loci associated with periodontitis, with some genetic elements overlapping between periodontitis and other inflammatory disorders like atherosclerotic cardiovascular disease [14]. Notably, the genetic contribution to periodontal disease may vary across different age groups, with younger individuals potentially experiencing a more pronounced genetic influence than older counterparts [14]. Despite genetic predispositions, maintaining stringent oral hygiene practices remains paramount in mitigating the risk of periodontal disease for all individuals [15]. This underscores the critical importance of adhering to regular and effective oral health routines, regardless of one's genetic predisposition, in staving off the development and progression of periodontal disease.

Environmental and Behavioral Factors

Environmental and behavioral factors wield considerable influence on the onset and progression of periodontal disease. Environmental factors, such as tobacco smoking and psychological stress, have the potential to disrupt the symbiotic equilibrium within the oral cavity, thereby contributing to the pathogenesis of periodontal disease [16]. Conversely, behavioral factors encompassing aspects like poor oral hygiene, smoking, alcohol consumption, and an unhealthy diet represent modifiable risk factors that significantly impact gum health [17]. This underscores the pivotal role of lifestyle choices in managing oral health. Extensive research underscores the profound impact of both environmental and behavioral factors on periodontal disease. It underscores the imperative for holistic comprehensive strategies to address these aspects to prevent and manage this prevalent oral health condition. Such approaches would entail addressing detrimental environmental influences and promoting positive behavioral changes to foster optimal gum health.

PE: clinical features and pathophysiology

Definition and Diagnostic Criteria

The definition and diagnostic criteria for PE entail the emergence of new-onset hypertension occurring after 20 weeks of gestation, accompanied by proteinuria or indications of end-organ impairment. Specifically, PE is confirmed when hypertension is present (systolic blood pressure ≥ 140 mmHg and/or diastolic blood pressure ≥ 90 mmHg) alongside proteinuria (exceeding 300 mg/24 hours or a urine protein/creatinine ratio of ≥0.3), or when there are signs of end-organ damage such as thrombocytopenia, compromised liver function, renal insufficiency, pulmonary edema, new-onset headache, or visual disturbances [18-20]. Severe manifestations of PE are identified by sustained severe hypertension (systolic blood pressure ≥ 160 mmHg and/or diastolic blood pressure ≥ 110 mmHg) in conjunction with indications or symptoms of end-organ impairment [20]. Notably, the diagnosis of PE may still be established even in the absence of proteinuria if there is new-onset hypertension accompanied by signs of end-organ damage [20]. Furthermore, eclampsia is typified by unexplained generalized seizures occurring in individuals diagnosed with PE, underscoring the critical importance of precise diagnosis and prompt intervention to mitigate risks posed to both the mother and fetus [21]. This emphasizes the urgency of accurate diagnostic assessment and timely management to ensure optimal maternal and fetal health outcomes.

Pathophysiological Mechanisms

The pathophysiological mechanisms underlying PE encompass a spectrum of factors, including defective placentation, placental ischemia, abnormal remodeling of spiral arteries, oxidative stress at the maternal-fetal interface, and an imbalance in angiogenic factors within the maternal circulation. These factors collectively precipitate endothelial dysfunction and subsequent end-organ damage [2,19,22-24]. A pivotal aspect contributing to the pathophysiology of PE involves the anti-angiogenic activity of proteins such as sFlt-1 (soluble fms-like tyrosine kinase-1) and sEng (soluble endoglin). These proteins are excessively produced in PE, leading to endothelial dysfunction and the involvement of multiple organs [24]. In early-onset PE, incomplete transformation of spiral arteries results in placental hypoperfusion and fetal growth restriction. Conversely, late-onset PE may involve altered spiral arteries without fetal growth restriction, suggesting distinct pathophysiological pathways for early and late-onset presentations [19]. Comprehending these intricate pathophysiological mechanisms is imperative for the development of diagnostic modalities and tailored therapeutic interventions aimed at managing and preventing the adverse outcomes associated with PE. Such understanding lays the foundation for the advancement of strategies aimed at mitigating the impact of this serious pregnancy complication on maternal and fetal health.

Maternal and Fetal Complications

PE poses significant maternal complications, ranging from organ system damage to potentially life-threatening conditions. These complications include pulmonary edema, seizures (eclampsia), renal failure, liver hematoma or rupture, and bleeding complications [25]. Eclampsia, a complication that arises in a portion of women with severe PE, can result in substantial maternal morbidity and mortality. Sequelae of eclampsia may include intracerebral hemorrhage, transient blindness, and cardiorespiratory arrest, with maternal mortality rates spanning from 0 to 14% [25]. Fetal complications associated with PE stem from impaired placental function, leading to inadequate oxygen and nutrient delivery to the fetus. These complications include intrauterine growth restriction, preterm birth, and in severe cases, stillbirth due to placental abruption. Infants affected by PE are at increased risk of long-term health issues, including learning disorders, cerebral palsy, epilepsy, sensory impairments (deafness and blindness), as well as metabolic and cardiovascular conditions such as diabetes, congestive heart failure, and hypertension [26]. These substantial maternal and fetal complications underscore the critical importance of early detection, vigilant monitoring, and appropriate management of PE to mitigate adverse outcomes. Implementing comprehensive strategies aimed at timely intervention and optimized care is essential in safeguarding the mother's and fetus's health and well-being.

Evidence of association between periodontal disease and PE

Epidemiological Studies

Multiple meta-analyses and systematic reviews have revealed a positive correlation between maternal periodontal disease and an elevated risk of PE [27,28,29]. One meta-analysis indicated that pregnant women exhibiting signs of periodontal disease had a 1.76-fold greater risk of PE compared to those without periodontal disease [27]. Nevertheless, not all studies have yielded statistically significant associations. Some investigations have reported no discernible link between maternal periodontal disease and the risk of PE [30]. Discrepancies in study definitions, diagnostic criteria, and methodological quality likely contribute to the inconsistent findings [27,28,30]. The relationship between periodontal disease and PE appears to be multifaceted, with certain studies suggesting a stronger association with severe periodontal disease [28,30]. A case-control study, for instance, identified a 3.32-fold increased risk of PE among individuals with severe periodontal disease [30]. Potential mechanisms underlying the link between periodontal disease and PE include inflammation and endothelial dysfunction [28,30]. However, further research is warranted to understand the underlying pathways involved comprehensively. Continued investigation into these mechanisms is imperative for elucidating the intricate relationship between periodontal disease and PE and informing the development of targeted preventive and therapeutic interventions.

Biological Plausibility

*Biological plausibility* denotes the coherence and viability of a proposed biological mechanism or relationship between two variables or conditions. In the context of the association between periodontal disease and PE, biological plausibility pertains to whether a logical and scientifically supported connection exists between periodontal disease and the development of PE based on biological mechanisms. Insights from provided sources shed light on the biological plausibility of the association between periodontal disease and PE. They discuss how periodontal disease, characterized by chronic inflammation and dysbiosis of the oral microbiota, may contribute to systemic inflammation and the release of inflammatory molecules that could potentially impact the placenta and precipitate PE [31]. Specific periodontal pathogens, notably P. gingivalis, have been implicated in eliciting inflammatory responses that may affect pregnancy outcomes [30]. Furthermore, the literature suggests that the inflammatory response associated with periodontal disease may trigger systemic effects capable of influencing the maternal-fetal interface, thus potentially leading to adverse pregnancy outcomes such as PE [32]. While certain studies have reported a positive association between periodontal disease and PE, conflicting results have been documented in others, underscoring the necessity for further research to comprehensively grasp the biological plausibility of this relationship [30]. Continued investigation into these mechanisms is crucial for elucidating the intricate interplay between periodontal disease and PE and informing the development of preventive and therapeutic strategies to improve maternal and fetal health outcomes.

Mechanistic Pathways

Periodontal pathogens can potentially enter the bloodstream and trigger an inflammatory response within the placenta [31]. Specific pathogens such as P. gingivalis can modulate the host immune system, promoting a shift in the oral microbiota from symbiotic to dysbiotic states, thereby fostering inflammatory disorders [31]. Inflammatory molecules generated in response to periodontal pathogens can disseminate via the bloodstream, eliciting an inflammatory reaction within the placenta [31]. Furthermore, heightened bleeding at periodontal sites may facilitate the hematological spread of periodontal pathogens and their byproducts, culminating in an immune or inflammatory response within the fetoplacental unit [31]. Additionally, periodontal pathogens have been observed to colonize the vaginal microbiota, potentially through gastrointestinal transit or oro-genital contacts, thereby contributing to the inflammatory processes associated with PE [31]. Since periodontal disease represents a state of chronic low-grade inflammation, it is conceivable that it contributes to the generalized inflammatory response pivotal in the pathogenesis of PE [33]. Indeed, studies have reported an association between periodontal disease and early-onset PE (occurring before 34 weeks of pregnancy) [33]. Various factors, including metabolic conditions, immunological changes, and fluctuations in progesterone and estrogen levels during pregnancy, can precipitate oral microbiota dysbiosis and heightened inflammation of periodontal tissues [34]. This dysregulation may exacerbate the progression of PE, underscoring the intricate interplay between oral health and pregnancy-related complications.

Mechanisms underlying the association

Inflammatory Pathways

The association between periodontal disease and PE hinges on inflammatory pathways that play a pivotal role in the pathogenesis of adverse pregnancy outcomes. Evidence suggests that periodontal inflammation can incite systemic inflammatory responses during pregnancy, thereby potentially predisposing individuals to conditions like PE [31,35,36]. Inflammatory mediators generated in response to periodontal pathogens can travel through the bloodstream, provoking an inflammatory reaction within the placenta and contributing to the onset of PE [31]. Moreover, certain periodontal pathogens, notably P. gingivalis, have been implicated in the pathogenesis of PE by their ability to activate inflammatory signaling pathways both locally and in extra-oral sites, including the placenta-fetal unit [36,37]. The hematological dissemination of periodontal pathogens from bleeding sites can precipitate immune and inflammatory responses within the fetoplacental unit. This underscores the significance of inflammatory pathways in bridging the gap between periodontal disease and adverse pregnancy outcomes such as PE [31]. The inflammatory pathways associated with periodontal disease may exert systemic effects during pregnancy, potentially contributing to the development of PE by eliciting inflammatory responses within the placenta and systemic circulation. Further research is warranted to elucidate the specific inflammatory markers and mechanisms underlying the relationship between periodontal disease and PE, thereby informing targeted interventions to mitigate the risk and severity of PE in pregnant individuals.

Endothelial Dysfunction

Endothelial dysfunction is a state where the endothelium, the inner lining of blood vessels, experiences impaired functionality, marked by a range of abnormalities such as vasoconstriction, heightened vascular permeability, thrombosis, and inflammation [38]. This condition represents a systemic pathological state of the endothelium, precipitating a cascade of detrimental effects, including vasoconstriction, vascular leakage, thrombosis, hyperinflammation, and a compromised antiviral immune response [38]. Linked to conditions like atherosclerosis, endothelial dysfunction may precede vascular pathology and contribute to the formation of atherosclerotic plaques through mechanisms such as enhanced adherence of monocytes and macrophages, infiltration of low-density lipoprotein (LDL), and oxidative stress [38]. The primary cause of endothelial dysfunction lies in the impaired bioavailability of nitric oxide, a pivotal mediator responsible for regulating vascular tone and oxidative stress within the endothelium [39]. Diminished nitric oxide levels can lead to vasoconstriction, heightening the risk of cardiovascular diseases, atherosclerosis, hypertension, and angina (chest pain) [39]. Management of endothelial dysfunction typically involves lifestyle modifications, medication regimens such as ACE inhibitors, calcium channel blockers, and statins, alongside behavioral changes aimed at mitigating risk factors [39]. Given its association with various cardiovascular complications, including atherosclerosis and coronary artery disease, a comprehensive understanding of the mechanisms and risk factors underpinning endothelial dysfunction is imperative for effective diagnosis, treatment, and management. This knowledge is instrumental in preventing adverse cardiovascular events and improving patient outcomes.

Immune Modulation

The concept of immune modulation involves the deliberate adjustment of the immune system to achieve therapeutic objectives, particularly in cancer therapy. Immune modulation strategies in cancer treatment are geared towards harnessing the patient's immune system to control, stabilize, and ideally eliminate tumors. This encompasses a spectrum of interventions designed to induce, amplify, attenuate, or prevent immune responses tailored to specific therapeutic goals [40]. In cancer therapy, immune modulation is pivotal in bolstering the body's immune response against malignant cells. Central to this approach are immune checkpoint antibodies, which target specific immunomodulatory receptors on T cells and other immune cells. Antibodies such as CTLA-4, PD-1, and PD-L1 blockers have demonstrated clinical efficacy across a spectrum of malignancies, broadening the application of immunotherapy to cancers traditionally deemed less responsive to conventional treatments [40]. Moreover, ongoing research explores novel immune checkpoint molecules, including agonists of co-stimulatory molecules like CD-137, OX40, and GITR. These agents aim to augment co-stimulatory signals on B and T cells, potentially resulting in tumor regression and enhanced therapeutic outcomes in advanced solid tumors. The field of immune modulation in cancer therapy holds considerable promise, leveraging the body's immune defenses to combat cancer. By targeting specific immune checkpoints and investigating innovative immune checkpoint molecules, researchers are advancing strategies to bolster immune responses against tumors and enhance treatment efficacy across various cancer types [41].

Microbiome Alterations

Microbiome alterations, particularly within the gut microbiota, have emerged as significant contributors to PE development. Research indicates that shifts in the gut microbiota composition can impact the risk of PE by instigating both local and systemic inflammation, potentially precipitating the onset of this pregnancy complication [42-44]. Specific microbial taxa linked with PE, such as Bifidobacterium, Collinsella, and Enterorhabdus, have been identified, demonstrating a causal association with PE and possibly exerting a protective role against this condition [42,44]. Moreover, dysbiosis of the gut microbiome in PE patients has been correlated with immune dysregulation, intestinal barrier dysfunction, and bacterial translocation to the intrauterine cavity, culminating in placental inflammation and impairing placental function [44]. Evidence suggests that the dysbiotic gut microbiome can trigger elevated blood pressure, proteinuria, and other adverse outcomes in both human and animal models, underscoring the potential impact of microbiome alterations on the pathogenesis of PE [44]. The perturbations observed in the gut microbiome, characterized by dysbiosis and alterations in specific microbial taxa, wield significant influence over the development of PE by modulating inflammatory responses, immune function, and placental health. Insight into these microbiome alterations holds promise for predicting, preventing, and managing PE in clinical practice. Continued research into the intricate interplay between gut microbiota and PE pathogenesis is crucial for advancing our understanding and improving clinical approaches to mitigate the risks associated with this pregnancy complication.

Clinical implications and management

Screening Protocols

The Basic Periodontal Examination (BPE) is a straightforward and swift screening tool for evaluating adult patients [45]. It entails probing the periodontal tissues to assess parameters such as bleeding on probing, plaque and calculus deposits, and pocket depths [46]. Utilizing a World Health Organization (WHO) Community Periodontal Index of Treatment Needs (CPITN) probe, the BPE assigns a score to each sextant based on the most affected site [47]. While the BPE offers guidance on further periodontal assessment and treatment required by the patient, it does not yield a definitive diagnosis [47]. Similarly, the Periodontal Screening and Recording (PSR) method shares similarities with the BPE and is employed for screening children and adolescents [47]. It involves probing the periodontal tissues and assigning a score to each sextant based on the highest score observed. For patients undergoing supportive periodontal therapy, annual full-mouth periodontal charting is recommended. This comprehensive procedure entails meticulously recording probing depths throughout the dentition, either manually using periodontal probes or using computerized probes. Full-mouth charting furnishes detailed information crucial for managing patients with periodontitis. Furthermore, salivary biomarkers, such as hemoglobin levels, can be leveraged for screening periodontal diseases. Salivary tests offer a non-invasive means of assessment and can aid in predicting the progression of chronic periodontitis. However, additional research is warranted to ascertain the accuracy and reliability of salivary screening tests [48].

Periodontal Treatment During Pregnancy

Periodontal treatment administered during the second trimester of pregnancy has shown promise in reducing adverse pregnancy outcomes, including rates of preterm low birth weight [35,49]. This treatment regimen typically involves nonsurgical procedures such as scaling and root planing, coupled with mouthwash using chlorhexidine solution, which has demonstrated positive effects in mitigating adverse outcomes [49]. It is advised that periodontal treatment for pregnant women be conducted during the second trimester when the fetus is in a stable state, thereby minimizing the risk of complications [50]. Early detection and intervention for periodontal disease during pregnancy are paramount to forestall adverse effects on both maternal and fetal health [50]. Nonsurgical periodontal treatment during pregnancy is generally regarded as safe and beneficial for the well-being of both mother and fetus [35]. Sustaining oral health throughout pregnancy is imperative, and a multifaceted approach is often more efficacious than relying on a singular intervention type [35]. To thwart the progression of periodontal disease, which can precipitate adverse pregnancy outcomes, pregnant individuals must uphold good oral hygiene practices and seek prompt dental care [50]. Dental professionals advocate for regular oral examinations before conception to address any existing oral infections and preempt the onset of periodontal disease during gestation [50].

Impact on Obstetric Outcomes

The impact of periodontal treatment on obstetric outcomes, particularly in pregnant women, has garnered considerable attention and research scrutiny. A meta-analysis concluded that administering scaling and root planing for periodontitis during pregnancy did not yield significant effects on obstetric outcomes, including preterm birth and low birth weight [51]. Despite initial speculation positing that treating periodontal disease could mitigate the risk of adverse pregnancy outcomes, subsequent large-scale trials failed to demonstrate any noteworthy benefits from treatment. This led to the consensus that screening for and treating periodontal disease during pregnancy may not substantially improve pregnancy outcomes [32]. Observational studies have lent support to an association between periodontitis and adverse pregnancy outcomes such as preterm birth, low birth weight, gestational diabetes mellitus (GDM), and PE [51]. However, questions have been raised regarding the effectiveness of periodontal treatment in reducing these adverse outcomes. Evidence has emerged suggesting that periodontal disease itself may not be directly linked to adverse pregnancy outcomes [32,49].

## Conclusions

In conclusion, this comprehensive review elucidates the significant association between periodontal disease and PE, shedding light on its epidemiological patterns, potential mechanistic pathways, and clinical implications. Epidemiological evidence consistently suggests a heightened risk of PE among pregnant individuals with periodontal disease, underscoring the importance of oral health in maternal-fetal outcomes. Mechanisms such as systemic inflammation, endothelial dysfunction, and immune dysregulation are proposed to underlie this association, though further research is warranted to elucidate these pathways definitively. Clinically, the findings advocate for heightened awareness among obstetricians regarding the oral health status of pregnant individuals, prompting consideration for routine periodontal screening as part of prenatal care. Moreover, interdisciplinary collaboration between obstetricians and periodontists is encouraged to optimize the management of periodontal disease during pregnancy and potentially mitigate the risk of PE. Future research should prioritize longitudinal studies to establish causality, mechanistic investigations to delineate specific pathways, and randomized controlled trials to evaluate the efficacy of periodontal interventions in preventing PE. By addressing these research gaps, we can advance our understanding of the complex interplay between oral health and obstetric outcomes, ultimately improving maternal and fetal health outcomes.
